# A Case of Bipolar Affective Disorder and Aspiration Pneumonia

**DOI:** 10.1155/2013/360348

**Published:** 2013-07-15

**Authors:** Alessandro Gerada, Gaetano Dell'Erba

**Affiliations:** Lucille Van Geest Centre, Thorpe Road, Peterborough, Cambridgeshire PE3 6DA, UK

## Abstract

Adults with mental illness are at a higher risk of aspiration pneumonia than the general population. We describe the case of a patient with bipolar affective disorder and two separate episodes of aspiration pneumonia associated with acute mania. We propose that he had multiple predisposing factors, including hyperverbosity, sedative medications, polydipsia (psychogenic and secondary to a comorbidity of diabetes insipidus), and neuroleptic side effects.

## 1. Background

Aspiration pneumonia is often a severe illness. There are multiple predispositions that increase the risk of aspiration, some of which are especially important in mental health patients. To the best of our knowledge, this is the first documented case report of recurrent life-threatening aspiration pneumonia separated in time. 

## 2. Case Presentation

We present, with the patient's consent, the case of a 63-year-old gentleman with longstanding bipolar affective disorder who presented hypomanic and went on to develop life-threatening aspiration pneumonia and nephrogenic diabetes insipidus. 

He had a long history of bipolar affective disorder extending back 25 years. Other comorbidities include asthma, type 2 diabetes mellitus, hypertension, and chronic kidney disease. In a hospital admission in 2009, he had presented in a state of mania and required rapid tranquilisation. He subsequently suffered from a severe aspiration pneumonia and hypernatraemia, necessitating intensive care treatment, tracheostomy, and PEG feeding due to dysphagia. He has since suffered from a nonspecific muscle weakness, the cause of which remains unclear. He was able to eat normally at home, and the PEG had been removed four months prior to admission. 

In this informal admission, he presented in a hypomanic state, with hyperverbosity, loud speech, disinhibition, and excessive drinking of milk. The likely trigger for this episode was the change from lithium to lamotrigine (25 mg bd) two months earlier by his community team, due to mildly impaired renal function. The other psychotropic medications on admission were promethazine 25 mg nocte, clonazepam 0.5 mg bd and sodium valproate 1 g bd. 

His renal function had improved since stopping lithium; eGFR on admission was 53, while other blood tests were unremarkable. Of note, sodium levels were 145 mmoL/L. 

On the ward, there were episodes of agitation, which were easily managed with verbal deescalation and oral lorazepam. A decision was taken to stop clonazepam, lamotrigine, and promethazine. Due to a long history of different antipsychotics in the past, a trial of asenapine was started at 5 mg bd. While his mental state remained largely unchanged, he started complaining of shortness of breath one week into the admission, which was later accompanied by chest pains, pyrexia, and oxygen saturations of 90%. He was transferred to an acute hospital with a diagnosis of aspiration pneumonia. Initial treatment was with intravenous fluids and antibiotics. His sodium was found to be deranged at 184 mmoL/L despite attempts at correction. A highly impaired swallow was observed, and he remained nil by mouth. All psychotropic medications were stopped during this deterioration of physical health. He made a good recovery after a period in intensive care, and his sodium was normalised with fluid replacement. 

## 3. Discussion

Our patient appears to have suffered two separate episodes of life-threatening aspiration pneumonia and hypernatraemia associated with manic episodes. The hypernatraemia responded to strict fluid management and improved in parallel with the patient's general condition. The patient likely suffered from nephrogenic diabetes insipidus despite being off lithium for over two months. Histological studies have revealed that chronic lithium use can lead to a chronic focal interstitial nephropathy which may not be reversible [1].

We do not have any evidence that our patient had impaired swallowing immediately prior to the episodes; indeed his PEG had been recently removed after a successful swallow assessment before the second episode. Our literature review reveals another case where a patient suffered from severe aspiration during mania [2]. They speculated that the cause may be hyperverbosity. A recent systematic review of 10 studies revealed that the frequency of dysphagia in adults with mental illness was 9 to 46%, compared to 6% in the general population [3]. Causes may be multifactorial, including behavioural and pharmacological. 

Patients with acute mania may be at a significantly increased risk of aspiration due to a combination of hyperverbosity, sedative medications, polydipsia (psychogenic and secondary to diabetes insipidus), and neuroleptic side effects. Our patient appears to have had recurrent aspiration pneumonia in association with manic episodes. There is no current guidance on interventions in managing dysphagia in adults with mental health illness [3]. 
